# Removal of Separated Endodontic K-File with the Aid of Hypodermic Needle and Cyanoacrylate

**DOI:** 10.1155/2016/3970743

**Published:** 2016-10-03

**Authors:** Luciana Maria Arcanjo Frota, Bernardo Almeida Aguiar, Maria Gerusa Brito Aragão, Bruno Carvalho de Vasconcelos

**Affiliations:** ^1^Post-Graduate Program in Dentistry, Federal University of Ceará, Fortaleza, CE, Brazil; ^2^School of Dentistry of Sobral, Federal University of Ceará, Campus Sobral, Sobral, CE, Brazil

## Abstract

A wide range of accidents might happen during the treatment of the root canal system, where the instrument separation is one of the most unpleasant occurrences. Several techniques have been developed to facilitate the removal of the fragments; however, they generally require specific devices that not always are available to the clinician. The aim of this case report is to present a simple alternative technique to remove from the root canals manual instruments fractured during the treatment. The case has its outline based on a 31-year-old patient who sought the clinic to have her maxillary first left premolar rehabilitated. The clinic and radiographic examinations revealed the need of endodontic retreatment and the presence of a fragment of a K-file instrument localized at the apical third of the palatine canal. The retreatment was initiated by the removal of the obturation materials followed by several unsuccessful attempts to take out the fractured instrument. Hence, it was chosen to perform the fragment removal using a hypodermic needle and cyanoacrylate adhesive. The fragment easily came out, which reinforces the technique adopted as a safe, simple, and low cost mean to solve the problem of fractured instruments using only items already present in the endodontic arsenal.

## 1. Introduction

Accidents might occur during the treatment of the root canal system, such as fracture of instruments, root perforations, and ledge formation, occurrences that increase the risks of failure of the endodontic treatment, since they reduce the effectiveness of the elimination of intracanal microorganisms, mainly of those that are located in inaccessible portions of the root canals [1]. One of the most unpleasant complications mentioned before is the fracture of instruments, as they are normally caused by cinematic movements wrongly applied on the instruments or by the use of deformed instruments that have lost their capacity of supporting the charge of an operation [2, 3].

The endodontic treatment is strictly dependent on the quality of the cleaning and shaping of the root canals. However, during these procedures, the risk of fracturing an instrument occurs mostly due to carelessness of the operator. A fractured instrument hinders the endodontic therapy, making it even more complex from the chemical mechanical preparation to the moment of the obturation of the canals, which negatively affects the long-term prognostic [4, 5]. Because of these iatrogenic aspects of the endodontic therapy, some clinicians end up finishing the procedure without informing the patients about such accidents. Clinical studies have reported an incidence of fractures ranking 0.39 to 5% among the cases of endodontic retreatment [2, 6].

The existence of a fragment inside the canals requires of the professional a detailed evaluation of the treatment options, immediately after the occurrence, or when planning a retreatment. It is extremely important for the clinician to know the complicating factors when removing a fragment from the root canals. They are the anatomy of the root canal system (RCS); the devices available to remove the fragment; the experience and ability of the professional to solve the problem; the localization, size, position, and diameter of the fractured instrument [2, 7]. Initially, three possibilities have to be considered when dealing with an instrument fractured inside the root canals. They are the choice of leaving the fragment into the canal, sealing it inside the canal, or removing it from the RCS [8]. It is essential to analyze the moment when the fracture happened due to the contamination risk, so the removal of the instrument will be dependent on the condition of the foraminal area. If the fractured instrument is removed from the RCS, the chances of success of the therapy increase, but this option requires complex procedures and specific materials that not always are available in the clinic for a great number of professionals. When the fragments are left into the canals, the prognosis is unclear, and this choice is still being discussed in the literature [7, 8].

Knowing that the removal of fractured instruments is one of the most difficult procedures in endodontics, but that this choice is essential to the success of the endodontic treatment, it is important to develop or adapt some techniques to facilitate such procedure. Several devices have been created, but none of them is completely effective to be used in all the cases. Moreover, there is no standardized protocol in the literature to be followed when it is necessary to remove a fractured instrument from the root canals [9].

Therefore, this study aims to present a simple alternative, secure, and low cost technique to remove fractured instruments from the root canals using only materials available in the clinic.

## 2. Case Report

**Figure 1:**
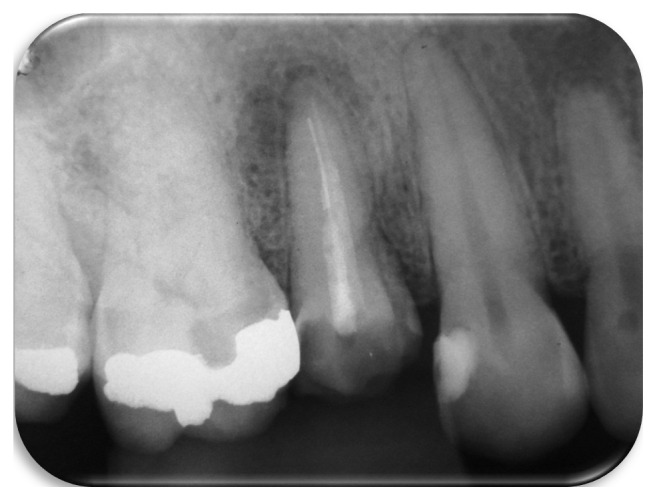
Initial periapical radiograph showing the fragment at the apical canal third.

A 31-year-old healthy woman patient sought the clinic to have her maxillary left first premolar rehabilitated. During the clinic examination, it was noticed that the mentioned tooth had undergone an endodontic treatment, which was exposed to the oral environment at the moment of the examination. The patient reported that the treatment had been in contact with the oral cavity for more than 6 months. It was decided to retreat the tooth, and to do so a periapical radiograph was performed in which the presence of a fragment was seen compatible with a hand-file of a size around 6 mm, localized at the apical third of the palatine root of the mentioned tooth (Figure 1).

**Figure 2:**
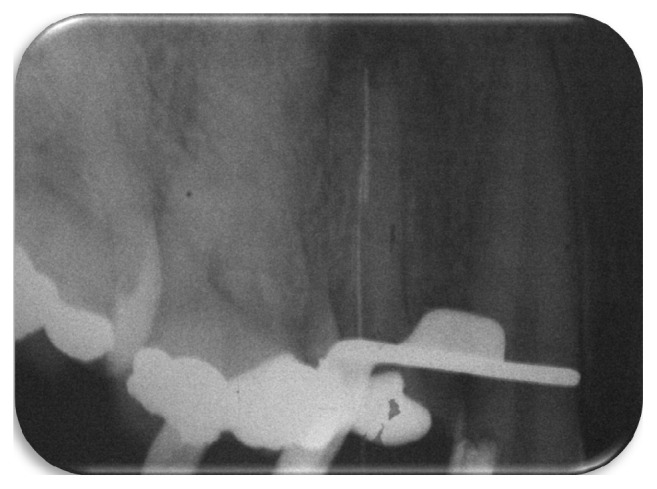
Periapical radiograph of the moment when trying to bypass the fractured instrument using manual files.

The endodontic treatment was initiated by the removal of the gutta-percha and of the endodontic sealer using the Gates-Glidden drills (#4, #3, and #2; Dentsply-Maillefer, Ballaigues, Switzerland) associated with repetitive irrigation of sodium hypochlorite 2.5%. Hand K-file instruments (Dentsply-Maillefer) were used for removing the filling material from apical thirds of canals. When the foraminal patency of the buccal canal was achieved, an attempt to bypass the fragment located in the palatine canal was achieved. An attempt of inserting manual instruments C-Pilot (#08, #10, and #15; VDW GbmH, Munich, German) in an apical direction tangentially to the fragment was performed. The instruments advanced 2 mm laterally to the fragment, but due to the difficulties related to the size of the fractured instrument and to the risk of causing a deviation in the original trajectory of the root canal, it was decided to stop this procedure (Figure 2).

**Figure 3:**
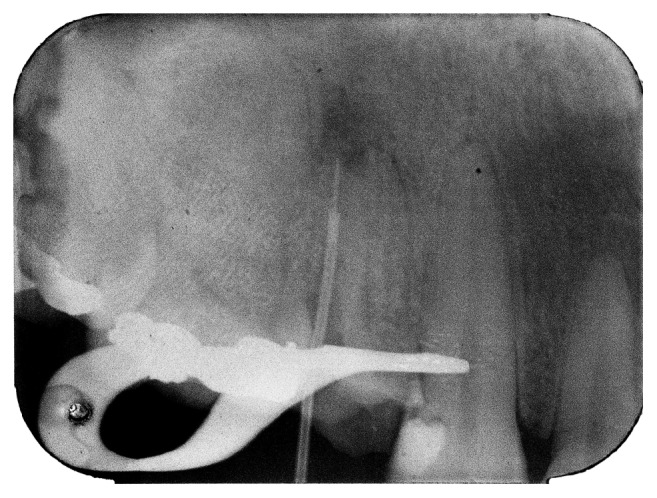
Periapical radiograph confirming the attachment of the fragment to the hypodermic needle.

Thus, it was decided to proceed with the attempt of removing the fragment using manual Hedstroem instruments (Dentsply-Maillefer) that were rotated to capture the fragment. When the capture was achieved, the Hedstroem instrument underwent repetitive coronal traction. This attempt was unsuccessful such as the first one, so it was decided to stop trying to perform it due to the risk of fracturing the instruments used. The lack of resoluteness of the techniques applied boosted the decision of using an alternative mechanic method to remove the fragment from the inside of the canal. To perform this method, a hypodermic needle (20 mm × 0.55 mm; Becton and Dickson, Curitiba, Brazil) and a cyanoacrylate adhesive (SuperBonder; Loctite, Itapevi, Brazil) were used. The active part of the needle was removed to make the attachment of the needle to the fragment easier. The needle was introduced into the canal, and when a tactile sensation of attachment was perceived, a periapical radiograph was taken to confirm that the two parts were perfectly attached to each other. After the confirmation (Figure 3), the cyanoacrylate adhesive was inserted in the aperture of the needle that was turned to the crown of the tooth using K-files and slight air jets.

**Figure 4:**
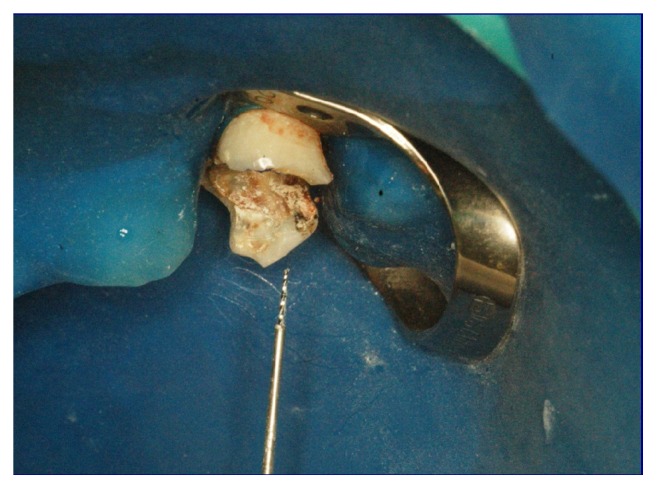
Photograph of the set hypodermic needle-fragment removed from the root canal.

After the polymerization time of 5 minutes, the hypodermic needle was rotated anticlockwise, allowing the unscrewing of the fragment (Figure 4) and its complete removal from the root canal.

**Figure 5:**
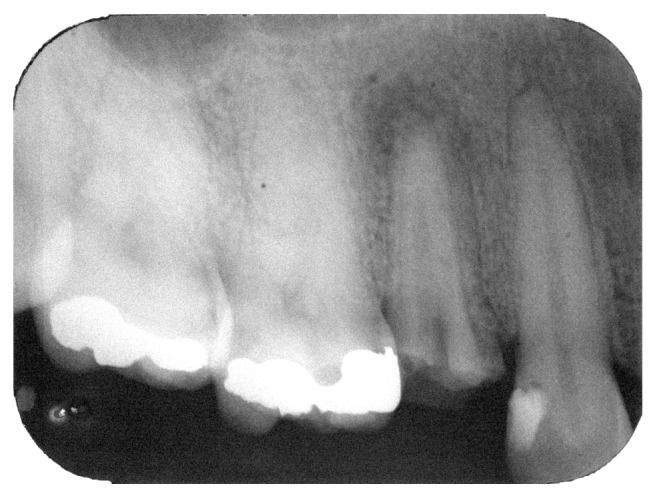
Periapical radiograph confirming the complete removal of the fragment.

**Figure 6:**
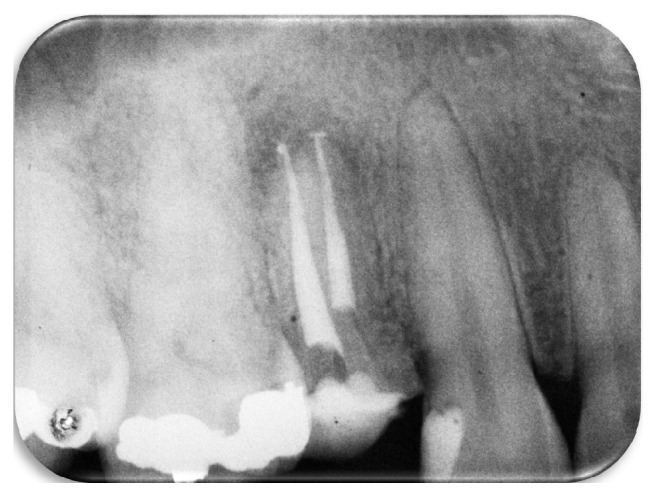
Periapical radiograph of the endodontic treatment completed.

A further radiographic examination revealed the successful removal of the fragment (Figure 5), which enabled the endodontic retreatment to be concluded satisfactorily (Figure 6).

## 3. Discussion

Procedural errors might occur during the treatment of the RCS as a result of factors that the clinician cannot control [3]. The facture of endodontic instruments is unpleasant occurrences that happen rather frequently in the endodontic clinic [1]. Overall, they are a result of cinematic movements incorrectly applied on instruments, or they are a consequence of the use of instruments that are already damaged, which increases the chances of fracture by torsion or cyclic fatigue [10, 11].

Several factors have to be considered before choosing to remove fractured instruments. The chances of success have to overweigh the possible complications [12]. Studies affirm that the success of the removal of the fragment is dependent on the type of instrument fractured, the anatomy of the canal, the type of tooth involved, and the technique applied to take the broken instrument out of the RCS [13, 14]. The impact of the size and of the irregularities of the canals on the success of the removal of the fractured instruments were highlighted by Hülsmann and Schinkel (1999), who pointed a higher success rate for anterior teeth with wide and straight canals than for posterior teeth canals, which are narrow and curved [12]. Suter et al. (2005) demonstrated a lower success rate for the cases when the fragment has to be removed from the apical third than when it has to be taken out of the medium or coronal third [7].

There are several techniques available in order for the clinicians to remove fractured instruments from the canals. Among them is the bypass followed by traction, which can or not be followed by the use of ultrasonic instruments [15]; the traction using the Masserann Kit [16], and the Canal Finder System. The first of the mentioned ones represents the association of procedures that are more often and widely used, but depending on the type and size of the fractured instrument, the technique might not be effective. However, the rest of the methods, in addition to requiring specific devices that make them the most expensive procedure, still show necessity of huge wear, compromising the tooth prognosis due to the excessive enlargement, and besides they are rarely used in areas of difficult access to canals [17].

In comparison to the other techniques above listed, the removal of a fractured instrument using a hypodermic needle associated with cyanoacrylate adhesive is a simple alternative technique and with low cost, because it does not require special devices, and uses routine materials in the dental clinic, and besides it is fast to be executed and does not require direct view of the light to the canal. Moreover, it was possible to verify one of its main advantages that it performs a small dental wear leading to minimum weakening of the tooth structure when compared with techniques described in the literature [3, 15, 16] significantly reducing the risk of subsequent fracture. However, this technique presents the difficulty of attaching the needle to the coronary portion of the fragment, which is not a problem when the clinician has appropriated training and hand ability.

An important aspect of the technique used in this case report that has to be highlighted is the security of the method, since it is not necessary to wear out tooth structure, or there is no need to try to bypass the fragment, which might result in perforations or deviation of the original trajectory of the canals. The removal of the fractured instrument allows the immediate access to the apical foramen, which consequently enables the clinician to perform the endodontic treatment satisfactorily.

The clinician needs to be aware of the techniques available, as well as of the several instruments that can be used to remove a separated fragment. The study of the localization of the fragment and the knowledge about the anatomy of the RCS are essentials to reduce a number of endodontic accidents, such as fractures. The technique used in this case report might be considered a conservative, secure, simple, and low cost option that can be performed by any professional in the day-to-day of the endodontic clinic.
